# Persistent Symptoms in Non-critical COVID-19 Patients at Two Months Follow-Up in a District Hospital: A Descriptive Cross-sectional Study

**DOI:** 10.31729/jnma.6440

**Published:** 2021-06-30

**Authors:** Bishal Tiwari, Manoj Ghimire, Gaurab Bhatta, Hemant Banstola, Bimala Tiwari, Anuradha Twayana, Karun Shrestha

**Affiliations:** 1 Beni Hospital, Myagdi, Gandaki, Nepal; 2 Gandaki Medical College, Pokhara, Gandaki Province, Nepal; 3 Kathmandu University School of Medical Sciences, Dhulikhel, Nepal; 4 Arthritis and Rheumatic Disease Treatment Centre, Lalitpur, Nepal

**Keywords:** *COVID-19*, *follow up*, *isolation*, *non-critical*, *persistent*

## Abstract

**Introduction:**

The corona virus disease 2019 is an infectious disease caused by severe acute respiratory syndrome coronavirus 2 belonging to corona viruses which are enveloped positive stranded RNA viruses. Non-critical coronavirus disease 2019 patients often lack follow up visits which has led to incomplete understanding of disease process. The aim of this study was to find out the prevalence of persistent symptoms in such patients during two months follow-up to a district hospital.

**Methods:**

This descriptive cross-sectional study was conducted in a district hospital from September 2020 to February 2021 among non-critical corona virus disease 2019 patients admitted to the isolation center of Nepal. Ethical approval was taken from the ethical review board of Nepal Health Research Council (reference number: 1707). Convenience sampling was done. Data was collected using a structured questionnaire. Data analysis was done using Statistical Package for the Social Sciences version 26. Point estimate at 95% Confidence Interval was calculated along with frequency and proportion for binary data.

**Results:**

Out of 132 patients, 66 (50%) (41.5-58.5 at 95% Confidence Interval) patients had persistent symptoms at two-month follow-up. Forty-eight (36.4%) patients showed one symptom, 15 (11%) had two symptoms, and 3 (2%) had two or more symptoms. The most frequent symptom reported was fatigue in 17 (13%), cough in 15 (11%), myalgia in 9 (7%), and headache in 9 (7%).

**Conclusions:**

The prevalence of persistent symptoms at two months follow up in our study was lower than findings from other international studies.

## INTRODUCTION

Coronavirus disease 2019 (COVID-19) is an infectious disease caused by severe acute respiratory syndrome coronavirus 2 (SARS-CoV-2) belonging to coronavirus family.^1,2^ Patients usually initially present with complain of fever, cough, dyspnea, sore throat, runny nose, smell or taste disorders. Several observational studies describe more one third of patients experiencing persistent physical and psychological symptoms as long COVID 19.^3-5^ Despite earlier data show shorter recovery time with mild disease, there is wide variability in duration for symptom resolution.^6^

Spectrum of severity in COVID-19 patient varies widely from asymptomatic to critical with majority being only mild symptomatic.^7^ There is huge gap of knowledge on clinical course of non-critical COVID-19 after being discharged from hospital.^8^

Thus, this study was done to find out the prevalence of persistence of COVID-19 symptoms among non-critical patients at two-month follow-up.

## METHODS

A descriptive cross-sectional study was conducted at the COVID-19 isolation hospital of Myagdi district, Nepal from September 2020 to February 2021. Ethical approval was taken from the Journal of Nepal Health Research Council (reference number: 1707). Non-critical COVID-19 patients, who do not develop any complications of COVID-19 and who do not need ICU or ventilatory support; fell under inclusion criteria of study. Exclusion criteria of study included patients deceased or admitted to the ICU, patients transferred to tertiary health care center for further management, and those who were unable to make follow up visit or unable to answer a phone within day 60 of symptoms onset. Subjects with medical emergency, vulnerable groups and children were also excluded. Convenience sampling was done and the sample size was calculated using the formula,

$$\begin{array}{ll} \text{n} & \text{= }{\text{Z}}^{\text{2}}\times \text{p}\times \text{q}/{\text{e}}^{\text{2}}\\ & \text{= }{\left(1.96\right)}^{\text{2}}\times 0.5\times (1-0.5)/{(\text{0.09)}}^{\text{2}}\\ & \text{= }119 \end{array}$$

Where,

n = minimum required sample size
Z = 1.96 at 95% Confidence Interval (CI)
p = prevalence taken as 50% for maximum sample size
q = 1-p
e = margin of error, 9%

The required sample size was 119. Adding a 10% nonresponse rate, we arrived at a sample size of 131. We included 132 patients in the study. All data were collected after informing participants about study objectives and significance of the study.

Data were entered and analyzed using the Statistical Package for the Social Sciences with version 26. Point estimate at 95% CI was calculated along with frequency and proportion for binary data.

## RESULTS

Of the 132 patients included in the study, the prevalence of persistent COVID-19 symptoms at two months follow up was 66 (50%) (41.5-58.5 at 95% CI). The remaining 66 (50%) were completely free from any COVID-19 related symptom. Fourty-eight (36.4%) had one symptom, 15 (11%) had two symptoms, three (2%) had two or more symptoms (Table 1).

**Table 1:** Number of symptoms present after COVID-19.

Number of Symptoms	n (%)
0	66 (50)
1	48 (36.4)
2	15 (11.4)
3	1 (0.8)
4	2 (1.5)

The mean duration of follow up was 60.9±1.035 with minimum of 59 and maximum of 65 days.

**Figure 1:**
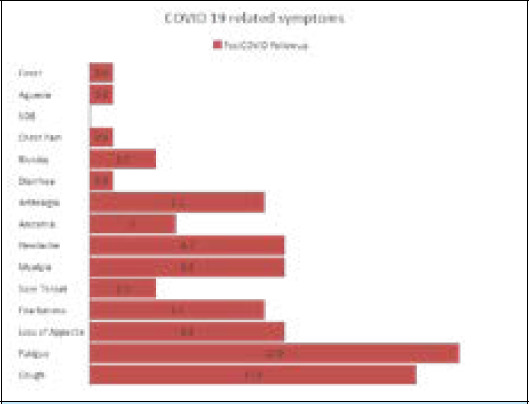
Persistent COVID-19 symptoms at two-month follow-up.

The most common symptoms in the order of their frequency were fatigue in 17 (13%), cough in 15 (11%), myalgia in 9 (7%), headache 9 (7%), loss of appetite in 9 (7%) (Table 2). None of the patients had shortness of breath or any signs or symptoms of acute illness (Figure 1).

**Table 2:** Persistent COVID-19 related symptoms present during follow-up.

S.N.	Symptoms	Absent n (%)	Present n (%)
1	Cough	117 (88.6)	15 (11.4)
2	Chest Pain	131 (99.2)	1 (0.8)
3	SOB^*^	132 (100)	0 (0)
4	Rhinitis	129 (97.7)	3 (2.3)
5	Sore Throat	129 (97.7)	3 (2.3)
6	Fatigue	115 (87.1)	17 (12.9)
7	Myalgia	123 (93.2)	9 (6.8)
8	Arthralgia	124 (93.2)	8 (6.1)
9	Loss of Appetite	123 (93.2)	9 (6.8)
10	Headache	123 (93.2)	9 (6.8)
11	Diarrhea	131 (99.2)	1 (0.8)
12	Fearfulness	124 (93.2)	8 (6.1)
13	Anosmia	128 (97)	4 (3)
14	Aguesia	131 (99.2)	1 (0.8)
15	Fever	131 (99.2)	1 (0.8)

* SOB-Shortness of Breath

**Figure 2:**
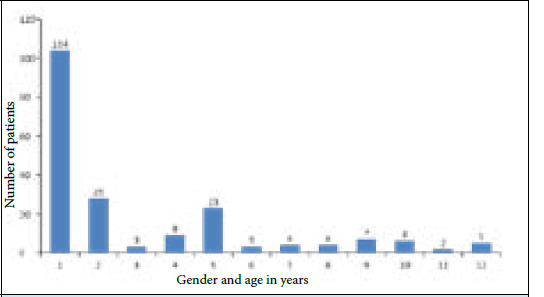
Gender and age-wise distribution of persistent COVID-19 symptoms.

There were 104 (78.8%) males and 28 (21%) females with a male: female ratio of 3.71:1. The mean age of the participants was 36±14 years. Persistent symptoms at two month follow up was more in age group of 2630 years (Figure 2).

## DISCUSSION

This study showed that patients who recovered from non-critical COVID-19 illness, 50 % of them reported at least one symptom particularly fatigue and cough, during two month follow up visit. The fundamental strength of our investigation was well documented follow up of patients with non-critical COVID-19 presentation at two months of disease course.

Clinical presentation of non-critical COVID-19 patients at the time of hospital admission was found to be similar to previous studies done in UK and Italy.^9,10^ A study done in Granada, Spain showed that 62.5% of patients hospitalized for COVID-19 infection report persistence of some symptom after a mean period of 50 days from discharge, dyspnea and asthenia being the most frequent, which resembles findings of this study.^11^ Report of WHO and China joint mission on COVID-19 stated that median time from illness onset to recovery is about 2 weeks for mild cases and 3 to 6 weeks with severe or critical disease.^12^

Regular follow up visit and care is essential for COVID-19 patients as some findings may be mild at initial presentation but persists during disease course hampering patient's quality of life. Subjective complaints of COVID-19 patients must be taken in medical care even after discharge from hospital and appropriate assessment must be made. Follow up visit at several intervals must be studied to analyze detail course of disease rather than only after two months. Similarly, follow up visit study must also take in account the psychosocial impact on patient's quality of life. Presence of other co morbid conditions and smoking history was not taken into consideration which has potential major role for symptoms persistence and duration of illness.

This was a descriptive cross-sectional study done in a single institution in a limited sample size. The findings may not be generalizable. Convenience sampling was done to enroll patients. Further studies must be done in a larger sample size. Also, we followed-up the patients at two months. We could not find out if the symptoms continue to persist, and if they do, until when.

## CONCLUSIONS

The prevalence of persistent symptoms at two months follow-up in our study was lower than findings from other international studies. With this study we found out that symptoms persisted even in those with mild initial presentation. This implies necessity of regular follow up visit and assessment for optimal patients care.
